# Pyoderma Gangrenosum after Breast Mammoplasty Surgery: A Case Report

**DOI:** 10.29252/wjps.10.2.103

**Published:** 2021-05

**Authors:** Chun Wa Fong, Sut Sin Tong, Monica Pon, Yun Fee Lai

**Affiliations:** 1 Department of Plastic and Reconstructive Surgery, Centro Hospitalar Conde São Januário, Macau SAR;; 2 Department of Internal Medicine, Centro Hospitalar Conde São Januário, Macau SAR

**Keywords:** Pyoderma gangrenosum, Mammoplasty, Breast reduction

## Abstract

Pyoderma gangrenosum (PG) is a rare inflammatory neutrophilic dermatosis, characterized by painful ulcerative, bullous, or pustular skin conditions. Pathergy is usually used to describe PG which refers to initiation or exacerbation of the disease after accidental or iatrogenic skin trauma. Diagnosis of postoperative PG is challenging not only due to its presentation mimics infectious wounds, but also because there are no standard laboratory parameters for diagnosis. Herein, we present a case of a 46-year-old female patient who had recurrent bilateral breast wound erythema, swelling, pain and necrosis after breast reduction mammoplasty at Centro Hospitalar Conde São Januário Macau SAR in 2018. We diagnosed her postoperative PG and successfully treated her with oral prednisolone with significant therapy response.

## INTRODUCTION

Pyoderma gangrenosum (PG) is a rare cutaneous ulcerative disease with an estimated incidence of 3 to 10 cases per million people per year^1^. It is usually related to pathergy and characterized by rapid progression of the painful necrolytic cutaneous ulcer with irregular violaceous undermined border. Postoperative PG has been reported which usually occurs within 7 to 14 d postoperatively. 

Here, we report the case of a 46-year-old female who had PG after a breast reduction mammoplasty. The emphasis of the current report is consideration of PG as one of the differential diagnosis of breast wound after operations. 

This study was approved by Medical Ethical Committee of Centro Hospitalar Conde São Januário, Macau SAR and the patient agreed with the publication of her case history and photographs.

## CASE REPORT

**Fig. 1:**
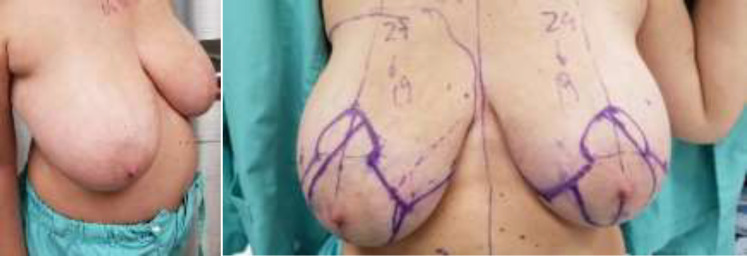
Patient with bilateral macromastia

A 46-year-old female presented with chronic back pain due to macromastia at Centro Hospitalar Conde São Januário, in 2018. Her bust size was 38F. Physical examination found asymmetric bilateral big breast which is larger on right side with ptosis (Figure 1).

Mammograms were benign. Her past medical history was unremarkable except for hepatic focal nodular hyperplasia, two times cesarean section and allergy to Penicillin. She was a smoker, about ten cigarettes per day. She denied alcohol abuse, usage of illicit drugs and history of diabetes.

In May 2018, under general anesthesia, bilateral reduction mammoplasty with superomedial pedicle, inverted T-technique was performed to reduce breast volume and achieve breast symmetry. The resected breast parenchyma was 399 gr on the left and 530 gr on the right. The estimated operative blood loss was 100 mL. 

**Fig. 2:**
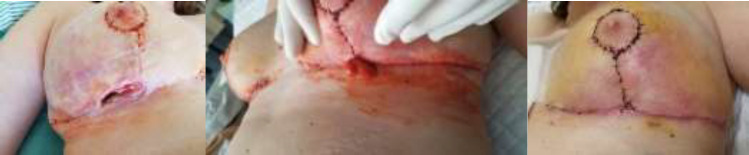
Seven days after bilateral breast reduction mammoplasty, there was dehiscence, erythema and discharge from the T-incisions

On the fifth postoperative day she developed pain on bilateral breasts and the seventh day had a low-grade fever (37.7 ℃). There was dehiscence and necrotic area at the junction of the T-incisions on the left breast, with erythema, swelling and drainage of liqueﬁed fat and purulent liquid bilaterally (Figure 2). In the following days, wound condition deteriorated and she had intermittent fever up to 38.3 ℃. Laboratory tests, including complete blood count, biochemical proﬁle, liver and renal function tests, were unremarkable except for high C-reactive protein level (5.91 mg/dL). Routine wound culture was negative. After wound care and empirical antibiotic usage (Ciprofloxacin and Clindamycin intravenously), bilateral breast wound condition seemed under control. She was discharged and received wound care at primary care. 

However, two weeks later, the patient presented again with local active infection signs at bilateral breast wounds. Therefore, she was re-admitted to ward for wound care and intravenous antibiotherapy (Levofloxacin and Clindamycin). 

Blood and skin cultures for anaerobic and aerobic bacteria and fungi were persistently negative. Breast ultrasound and MRI showed swelling of breast tissue and skin without abscess formation. In July 2018, debridement of bilateral breast wounds and excision of fistulas was performed. Pathology of left breast wound showed breast tissue exhibiting foci of aggregate of neutrophils, lymphocytes, plasma cells, histiocytes, and foreign body type giant cells. On the seventh day postoperatively, the patient developed fever up to 38.5 ℃ with local infection signs at bilateral breast wounds again. Cefuroxime and Clindamycin were given but without clinical improvement. Due to unsuccessful treatment with antibiotic, wound care and debridement, we considered PG and she was started on oral prednisolone 60 mg per day. 

**Fig. 3:**
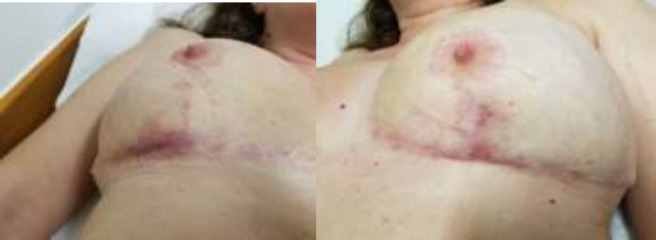
Bilateral breast wound healing after immunosuppressive therapy

Rapid clinical improvement confirmed the diagnosis. Breast pain, redness, and hotness largely resolved in three days after steroid initiated. Oral steroids were subsequently tapered in the following six-month period. The patient’s bilateral breast wounds healed, without signs of relapsed inflammation or infection (Figure 3).

 She kept regular follow up in outpatient clinic and possible related comorbidities such as rheumatoid arthritis, inflammatory bowel disease were excluded, as immune profile and colonoscopy were unremarkable.

## DISCUSSION

PG, first described by Dr. Brocq in 1916, is an uncommon non-infectious ulcerative skin disorder. Incidence of PG is estimated in approximately 3 to 10 in million people annually^1^. PG occurs predominantly in middle-aged women, with average age of 40 to 60 yr old. Etiology of PG remains unclear. Neutrophil function abnormalities, genetic variations and dysregulation of innate immune system contribute to the pathogenesis of PG^2^. Common comorbidities associated with PG are inflammatory bowel disease, rheumatoid arthritis, and myeloproliferative disorders^3^. However, recent clinical review found that 64% of patients with postoperative PG had no such underlying disease as in our described case^4^.

Base on clinical manifestations, PG is divided into ulcerative, bullous, pustular and vegetative subtypes. Ulcerative subtype is the classic one^5^. Our described case fits in the pustular subtype. 

Due to the nonspecific result in clinical findings and laboratory tests, early diagnosis of PG is challenging. It is often misdiagnosed as postoperative wound infection, cellulitis, or necrotizing fasciitis. Early diagnosis of PG is dependent on high suspicion and recognition. According to a systemic review about PG after breast surgery, the median time from initial presentation to correct diagnosis was on average 12.5 d, ranging from 2 d to 1095 days^6^. In general, PG occurs approximately 7 d after breast surgery presenting with infective signs over the surgical wound. There are a number of points worth noting in breast surgery complicated with PG. In literature review of series of cases showed that the lesion in PG is nipple sparing ^3^^,^^5^^,^^7^ and breasts are affected symmetrically. Intense pain out of proportion at breast examination is also one of the clues of PG. Su et al. suggested rapid progression in 1-2 cm daily or 50% monthly exacerbation in necrotic ulcer after breast surgery as diagnostic criteria of PG^8^. Failure of antibiotics and debridement combined with progressive surgical site inflammation is of decisive importance in the diagnosis of PG. Duval advocated the possibility of PG if clinical improvement is not achieved after 48 h of wide spectrum antibiotics^2^. Laboratory tests and wound culture related to PG are unspecific^3^^,^^5^. Biopsy of ulcer edge yielding a neutrophilic infiltrate, which is consistent with our case, appears to be useful diagnostic criteria of PG^8^. 

Immunosuppression with corticosteroids is the mainstay treatment of PG. A high dosage of systemic steroids initially (oral prednisolone 1mg/kg/day) and subsequent taper down schedule over 4 to 6 wk is recommended. Cyclosporine (2-5mg/kg daily) is an alternative management but the clinician must be cautious of renal toxicity and hypertension^8^. Surgical debridement is controversial since deterioration following surgical treatment was described^9^. It is consensual to avoid surgery in such group of patients. 

## CONCLUSION

Pyoderma gangrenosum of the breast is a rare disease. Definite diagnosis is challenging. There must be a high suspicion by clinician after exclusion of infection, cellulitis, necrotizing fasciitis or specific pathological result. Effective treatment is achieved with steroids or other immune suppressant drugs.

## CONFLICT OF INTEREST:

None.

## FUNDING:

None.
